# Salivary Periodontopathic Bacteria in Children and Adolescents with Down Syndrome

**DOI:** 10.1371/journal.pone.0162988

**Published:** 2016-10-11

**Authors:** Camila Faria Carrada, Flávia Almeida Ribeiro Scalioni, Dionéia Evangelista Cesar, Karina Lopes Devito, Luiz Cláudio Ribeiro, Rosangela Almeida Ribeiro

**Affiliations:** 1 School of Dentistry, Federal University of Juiz de Fora, Juiz de Fora, Minas Gerais, Brazil; 2 Department of Pediatric Dentistry, School of Dentistry, Federal University of Juiz de Fora, Juiz de Fora, Minas Gerais, Brazil; 3 Department of Ecology and Molecular Biology of Microorganisms, Institute of Biological Sciences, Federal University of Juiz de Fora, Juiz de Fora, Minas Gerais, Brazil; 4 Department of Dental Clinic, School of Dentistry, Federal University of Juiz de Fora, Juiz de Fora, Minas Gerais, Brazil; 5 Department of Statistics, Institute of Exact Sciences, Federal University of Juiz de Fora, Juiz de Fora, Minas Gerais, Brazil; University of the Pacific, UNITED STATES

## Abstract

**Objective:**

To assess and compare salivary periodontopathic bacteria between groups of Down syndrome and non-Down syndrome children and adolescents.

**Materials and Methods:**

This study included a sample of 30 Down syndrome children and adolescents (G-DS) and 30 age- and sex-matched non-Down syndrome subjects (G-ND). Clinical examination determined the gingival bleeding index (GBI) and plaque index. Unstimulated whole saliva samples were collected from all participants. The fluorescence *in situ* hybridization (FISH) technique identified the presence and density of eight periodontopathic bacteria in saliva. The statistical analysis included chi-square and Mann-Whitney *U* tests.

**Results:**

In the G-DS group, bleeding on probing was more frequent (*p* = 0.037) and higher densities of *Campylobacter rectus* (*p* = 0.013), *Porphyromonas gingivalis* (*p* = 0.025), *Treponema denticola* (*p* = 0.026), *Fusobacterium nucleatum* (*p* = 0.013), *Prevotella intermedia* (*p* = 0.001) and *Prevotella nigrescens* (*p* = 0.008) were observed. Besides, in the G-DS, the densities of bacteria from the orange complex were significantly higher in the age group 3–7 years for *F*. *nucleatum* (*p* = 0.029), *P*. *intermedia* (*p* = 0.001) and *P*. *nigrescens* (*p* = 0.006). *C*. *rectus was* higher in the age group 8–12 years (*p* = 0.045).

**Conclusion:**

The results showed that children and adolescents with Down syndrome have higher susceptibility to periodontal disease and number of periodontopathic bacteria.

## Introduction

Down syndrome (DS) is a genetic disorder that results from a trisomy on chromosome 21 and is present in approximately 1 in 600 to 1 in 1,000 live births [1]. Some reports have described a high prevalence of periodontal disease in children with DS, in which changes in the gingival tissue are frequent and occur early in life [2–6]. The increased prevalence and severity of this pathology in individuals with DS can be attributed to factors such as motor difficulty in performing oral hygiene, immune deficiency and the early and enhanced colonization of the oral cavity with periodontopathic bacteria [6].

Periodontal diseases are a group of conditions that affect the gingiva, periodontal ligaments, cementum, alveolar bones and tissue structures that support the teeth [7]. The disease begins with the growth of bacteria in the region of the gingival sulcus, which is especially colonized by gram-negative, anaerobic and microaerophilic bacteria that accumulate in an organized manner within the plaque. Some of the major periodontopathic bacteria found in the gingival sulcus include *Aggregatibacter actinomycetemcomitans; Porphyromonas gingivalis; Prevotella nigrescens; Fusobacterium nucleatum; Treponema denticola; Campylobacter rectus; Prevotella intermedia;* and *Tannerella forsythia* [8–10]. The orange complex, made up of *F*. *nucleatum*, *P*. *intermedia*, *P*. *nigrescens* and *C*. *rectus*, and the red complex, composed of *P*. *gingivalis*, *T*. *forsythia* and *T*. *denticola*, are the main etiologic agents of periodontal disease responsible for chronic periodontitis. The presence of these microorganisms is associated with the occurrence of bleeding during probing and other clinical manifestations of periodontal disease [11].

The presence of periodontopathic bacteria is critical to the development of periodontal disease, regardless of the immunological changes associated with DS [12]. Therefore, investigating the colonization of periodontal bacteria in children and adolescents with DS is particularly important for assessing the risk of developing periodontal disease and for effectively preventing and treating the disease. In a previous study on adolescents with DS, an altered microbial composition of subgingival plaque was observed, with a higher frequency of *A*. *actinomycetemcomitans*, *Capnocytophaga* and *P*. *gingivalis* detected [13]. Antibody titers for *P*. *gingivalis*, *P*. *intermedia*, *T*. *denticola*, *F*. *nucleatum*, *Selenomonas sputigena*, *A*. *actinomycetemcomitans* and *Streptococcus mitis* were positively correlated with clinical manifestations of periodontal disease in the primary dentition of children with DS [5]. Other studies have suggested that children with DS experience a very early colonization of various periodontopathic bacteria, with a higher prevalence of species such as *P*. *gingivalis*, *T*. *forsythia* and *T*. *denticola* [2]. In the subgingival microbiota of children, adolescents and young adults (8–28 years of age) with DS, the presence of *T*. *forsythia* and *Actinomyces naeslundii* was observed in all age groups; at older ages, patients presented with *P*. *gingivalis*, *A*. *actinomycetemcomitans*, *C*. *rectus*, *E*. *corrodens*, *P*. *intermedia*, *P*. *nigrescens*, *C*. *sputigena* and *P*. *micros* [6]. However, in addition to the identification of bacteria present, the amount of bacteria should be investigated due to increases in bacterial virulence and, hence, in the risk of developing periodontal disease.

Molecular microbiology techniques can provide rapid screening tools, thus providing important diagnostic approaches in the practice of preventive dentistry [8]. However, the majority of these techniques provide qualitative results indicating the presence or absence of microorganisms or semi-quantitative results that are obtained via DNA and RNA amplification [2]. The fluorescent *in situ* hybridization (FISH) technique provides information about the morphology, number and spatial distribution of various microorganisms [14], including periodontopathic bacteria [15–17]. Therefore, the aim of this study was to both qualitatively and quantitatively evaluate eight species of periodontopathic bacteria in the saliva of children and adolescents with and without Down syndrome.

## Materials and Methods

### Study design and sample characteristics

This observational cross-sectional study was approved by the Ethics Research Committee of the University Hospital of the Federal University of Juiz de Fora (Protocol No. 383/2011). Parents of children and adolescents who met the inclusion criteria provided consent for their children to participate in the study by signing a written informed consent form (ICF).

### Sample

Thirty children and adolescents with DS who were monitored by the Association of Parents and Friends of the Exceptional (APAE) were selected (G-DS), and thirty children without DS (G-ND) were selected among individuals in the same age group who were enrolled in an educational institution at Juiz de Fora, state of Minas Gerais, Brazil. To be included in the study, participants with or without DS were required to be between the ages of 3 and 12 with primary or mixed dentition. The subjects’ parents completed a health questionnaire that included information about systemic health. None of the participants in either group presented with other medical conditions known to affect periodontal status (e.g., diabetes mellitus) or were taking medications known to influence periodontal status. For the purposes of calculating ages, the last birthday was considered. Children and adolescents with Down syndrome were required to have a karyotype-confirmed diagnosis included in the APAE documentation and could not present with intellectual disabilities that precluded clinical examination. Children and adolescents who were undergoing orthodontic treatment and/or were being treated with antimicrobial drugs were excluded. Participants were divided into two groups as follows: the DS group (G-DS)–children and adolescents with DS; and the ND group (G-ND)–children and adolescents without the syndrome.

### Periodontal status

After data collection, the periodontal condition was evaluated by gingival bleeding on probing [18] and the modified version of the Silness and Löe plaque index (1964) [19]. For each participant, dentition was divided into six sextants called upper right, upper anterior, upper left, lower right, lower anterior and lower left. To represent each sextant, index teeth adopted in previous studies were examined, including the following primary teeth: upper right central incisors and lower left central incisor (51 and 71, respectively) and the first and second molars in each sextant (54, 55, 64, 65, 74, 75, 84, 85). When one of these teeth was missing, it should be replaced by its permanent successor or another tooth from the same sextant [20].

Bleeding on probing was recorded ten seconds after probing the mesial, distal, buccal, palatine and lingual surfaces of the index teeth using a periodontal probe (PCP-UNC 15, Hu-Friedy^®^, Chicago, IL, USA) according to a dichotomous pattern–presence (+) or absence (-) to calculate the Gingival Bleeding Index (GBI) proposed by Ainamo and Bay (1975), a dichotomous index to evaluate gingival inflammation. To calculate the prevalence of gingivitis in the groups, the presence of at least one bleeding area was adopted as the diagnostic criterion [18].

The plaque index was evaluated using a periodontal probe (PCP-UNC 15, Hu-Friedy^®^, Chicago, IL, USA) and a dental mirror. The presence of plaque on the buccal, lingual, mesial and distal surfaces of the index teeth was recorded according to the Silness and Löe plaque index (1964) [19], in which scores are assigned from 0 to 3 as follows:

- 0 –no plaque;
- 1 –a film of dental plaque adhering to the free gingival margin;
- 2 –moderate accumulation of dental plaque at the gingival margin; and
- 3 –abundant dental plaque in the gingival margin.

Before data collection, a single dental examiner (FARS) was trained by a specialist in pediatric dentistry (i.e., the gold standard–RAR–to ensure consistency in the bleeding during probing index). Six non-Down syndrome children (10% of the total sample) were previously examined twice with intervals of 7 days. The Cohen’s Kappa coefficients were *ĸ* = 0.93 for the intra-examiner agreement and *ĸ* = 0.92 for the inter-examiner agreement.

### Microbiological analysis

Unstimulated saliva samples were collected from the floor of the mouth of each volunteer after clinical examination using a disposable plastic Pasteur pipette (Qingdao AMA Co., Ltda, Shandong, China). Samples were collected from 8:00 a.m. to 12:00 noon and at least one hour after eating, brushing teeth or rinsing the mouth [21,22].

After collection, an automatic pipette was used to transfer 180 μL of saliva to a microcentrifuge tube containing 20 μL of 20% paraformaldehyde solution. The samples were maintained under refrigeration and were transported immediately to the Laboratory of Ecology and Molecular Biology of Microorganisms at UFJF. The fixed samples were stored at -20°C for subsequent microbiological analysis.

The identification and quantification of oral microorganisms were determined using the FISH technique. The samples were fixed in 20% paraformaldehyde (2% final concentration) and filtered through a 0.22 μm Millipore polycarbonate white filter. 16S rRNA nucleotide probes (Operon Technologies Inc., USA) were used and marked with Cy3 fluorochrome (Indo-carbocyanine) to identify oral microorganisms (Table 1).

**Table 1:** Oligonucleotide probes, 16S rRNA (Operon Technologies^®^) marked with Cy3 fluorochrome for the identification of oral microorganisms.

Probe	Specificity	Probe sequence (5’–3’)	% FA*	NaCl (mM)
**ACAC** [23]	*Aggregatibacter actinomycetemomitans*	TCCATAAGACAGATTC’	30	112
**B/TAFO** [23]	*Tannerella forsythia*	CGTATCTCATTTTATTCCCCTGTA	30	225
**CARE** [24]	*Campylobacter rectus*	TTAAACTTATGTAAAGAAG	20	80
**POGI** [23]	*Porphyromonas gingivalis*	CAATACTCGTATCGCCCGTTATTATTC	30	112
**TREII** [25]	*Treponema denticola*	GCTCCTTTCCTCATTTACCTTTAT’	30	56
**FUS664** [26]	*Fusobacterium nucleatum*	CTTGTAGTTCCGC(C/T)TACCTC	40	112
**Pint649** [27]	*Prevotella intermedia*	GCCGCCRCTGAASTCAAGCC	40	56
**Png657** [27]	*Prevotella nigrescens*	TCCGCCTGCGCTGCGTGTA	40	112

*Percentage of formamide (FA) in hybridization solution.

Subsequently, samples were stained with 4’,6-diamidino-2-phenylindole (DAPI) to quantify the total bacterial density. A negative control probe with no specificity for any bacterial group (5’-CCTAGTGACGCCGTCGAC-3') was used. A mix of the three positive control probes was also used–EUBI (5’-GCTGCCTCCCGTAGGAGT-3'), EUBII (5’-GCAGCCACCCGTAGGTGT-3') and EUBIII (5’-GCTGCCACCCGTAGGTGT-3') [28,29]. The filters were then divided into ten parts, one for each specific probe, the ninth part for the positive probe and the tenth part for the negative probe. Each piece of filter was placed on a glass slide coated with parafilm and was covered with 40 μL of hybridization solution with a final concentration of 2.5 ng/μL of the oligonucleotide probe. The hybridization solution was composed of 0.9 M NaCl, 20 mM Tris-HCl (pH 7.4), 0.01% sodium dodecyl sulfate (SDS) and a concentration of formamide that was specific for each specie (Table 1). The sample was incubated in a heater at 42°C overnight. After hybridization, the sample was transferred to a washing solution containing 20 mM Tris-HCl (pH 7.4), 5 mM EDTA, 0.01% sodium dodecyl sulphate and a NaCl concentration suitable for the specific probe (Table 1). Then, the sample was incubated at 48°C for 15 minutes. The bacterial cells were stained with DAPI so that the count of total bacterial density could be performed. Each piece of filter was immersed in 80% ethanol (v/v) three times and then allowed to dry. Finally, the slide was mounted using glycerol and Vectashield (Vector Laboratories Inc., Burlingame, CA, USA) at a ratio of 4:1.

The counting of the bacterial cells was carried out in ten random fields with Olympus BX60 epifluorescence microscope equipped with a 41007 filter for the Cy3 marker and a 31000 filter for DAPI at a magnification of 1000x. The results found for the hybridized samples using a probe without specificity were discounted from the densities of the specific probes. The total density of microorganisms was calculated by counting the cells stained with DAPI.

Cells in ten random fields were counted by a single researcher (CFC) trained by an experienced researcher (DEC). The percentage of each species with respect to total bacterial cell counts was calculated. The results were expressed as cells/mL.

### Statistical analysis

The data were organized in a database using the Statistical Package for Social Sciences (SPSS) version 15.0 for Windows. Categorical variables were described as a frequency distribution. Descriptive measures (mean, median, standard deviation, and minimum and maximum values) were used to describe the continuous variables for the surveyed microorganisms. The chi-square test was used for the analysis of age, gender and bleeding on probing variables. The Mann-Whitney *U* test for independent samples was used to compare the plaque index and the salivary densities of the tested bacteria between the groups [30]. In all analyses, a significance level of 5% was adopted.

Based on a previous study [16], a sample size of 30 participants in each group would be required to provide a 99% statistical power to identify a significant difference between groups. The data from this previous study indicated a standard deviation of 0.41 in the DS group, a standard deviation of 0.30 in the ND group and a mean difference of 0.43 in the number of *P*. *gingivalis* microorganisms between the groups.

## Results

The total sample included 60 children and adolescents aged 3–12 years living in the city of Juiz de Fora. Table 2 outlines the characteristics of the participants according to age and gender.

**Table 2:** Sample characteristics with respect to age and gender.

Variables	Total sample (N = 60)	G-DS (N = 30)	G-ND (N = 30)	*p-value*
Age (years)				
Mean ± standard deviation (years)	6.95 ± 2.38	6.37 ± 2.50	7.53 ± 2.15	0.057 ^ns^
Variation (years)	3–12	3–12	4–12	
Gender (n / %)				
Male (n / %)	31/51.70	17/56.70	14/43.70	0.606 ^ns^
Female (n / %)	29/48.30	13/43.30	16/53.30	

ns–difference not significant—Chi-square test.

Tables 3 and 4 present periodontal data that were obtained during clinical examination and evaluated by the gingival bleeding index and the plaque index. The G-DS group contained a significantly higher percentage of children and adolescents with bleeding on probing compared with the G-ND group (*p* = 0.037 –Table 3) (S1 Table). The mean plaque index did not differ between groups (*p* = 0.516 –Table 4) (S2 Table).

**Table 3:** Results of the gingival bleeding index.

Gingival bleeding index	G-DS (N = 30)		G-ND (N = 30)		*p-value*
	N	%	N	%	0.037*
**+**	11	36.70	4	13.30	
**-**	19	63.30	26	86.70	

* Significant difference–Chi-square test.

**Table 4:** Results of the Silness and Löe plaque index (1964).

G-DS		G-ND		*p–value*
Mean (Standard deviation)	Mean rank	Mean (Standard deviation)	Mean rank	0.516^ns^
0.49 (0.44)	31.92	0.43 (0.44)	29.08	

ns—Not significant difference—Mann-Whitney *U* test.

**Fig 1:**
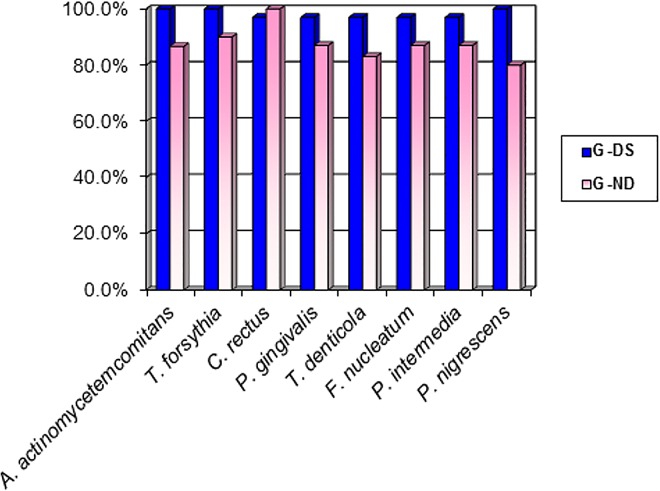
Frequency of children and adolescents from DS and ND groups with the evaluated periodontal bacteria.

Fig 1 shows the frequency of different types of periodontopathic bacteria in children and adolescents from the DS and ND groups. The frequency of periodontopathic bacteria in both groups combined ranged from 80 to 100%.

The descriptive measures of the density of eight species of bacteria in saliva (cells/mL x 10^8^) obtained in both groups are shown in Table 5 (S3 Table). The Mann-Whitney *U* test revealed a higher density of *C*. *rectus*, *P*. *gingivalis*, *T*. *denticola*, *F*. *nucleatum*, *P*. *intermedia* and *P*. *nigrescens* in saliva from participants in the G-DS group (S4 Table). Interestingly, the total density of bacteria was not significantly different in participants in the DS versus the ND group. The specific bacteria analyzed made up 12.20% of the total bacteria in the SD group and 10.52% of the total bacteria in the ND group (obtained by DAPI staining).

**Table 5:** Descriptive measures and results of Mann-Whitney *U* test (mean rank) for comparison of bacterial density (cells/mL X 10^8^) in the saliva of children and adolescents from G-DS and G-ND.

Bacteria	Median		Minimum		Maximum		Mean rank		*p-value*
	G-DS	G-ND	G-DS	G-ND	G-DS	G-ND	G-DS	G-ND
*A. a.*	8.77	5.85	1.35	0	40.05	85.05	34.78	26.22	0.057^ns^
*T. forsythia*	12.55	14.40	0	2.70	76.50	57.60	29.27	31.73	0.584^ns^
*C. rectus*	13.50	6.75	5.00	0	50.40	76.10	36.12	24.88	0.013*
*P. gingivalis*	14.40	10.80	2.70	0	57.60	50.40	35.55	25.45	0.025*
*T. denticola*	10.12	3.60	0	0	37.35	85.60	35.52	25.48	0.026*
*F. nucleatum*	11.70	4.72	0	0	83.70	245.70	36.12	24.88	0.013*
*P. intermedia*	12.15	3.60	0	0	82.80	90.00	37.88	23.12	0.001*
*P. nigrescens*	8.77	5.85	1.4	0	107.1	85.05	36.50	24.50	0.008*
Total (DAPI)	1052.55	1141.00	492.75	878.40	1489.05	2282.40	26.70	34.30	0.092^ns^

* Significant difference—Mann-Whitney *U* test.

ns—Not significant difference—Mann-Whitney U test.

**Fig 2:**
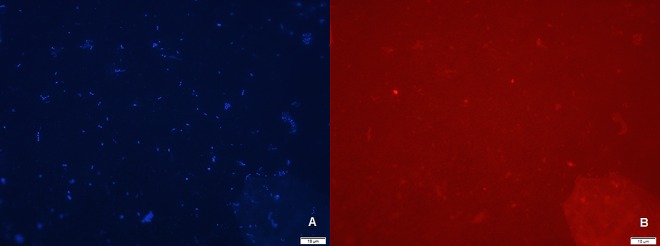
Illustration of Fluorescence *in situ* hybridization (FISH) technique: (A) the total microorganisms (DAPI) in the DS group and (B) the same field of view with targeted *F*. *nucleatum* cells (FUS664 probe). The scale bar indicates 10 μm.

Some species were not detected in the current study. Fig 2 shows a representative illustration of the total microorganisms (DAPI) in the DS group and the same field with targeted *F*. *nucleatum* cells (FUS664 probe).

Table 6 presents the comparisons between means of bacterial densities from orange and red complexes in the saliva of children and adolescents from the DS and ND groups divided into two age groups. The Mann-Whitney *U* test revealed a statistically significant difference in the density of bacteria from the orange complex, namely, *P*. *intermedia* (*p* = 0.001), *F*. *nucleatum* (*p* = 0.029), and *P*. *nigrescens* (*p =* 0.006) in samples from participants in the 3- to 7-year-old age range, and *C*. *rectus* (*p* = 0.045) in samples from the participants in the 8- to 12-year-old age range. The values were higher in the G-DS group.

**Table 6:** Result of the Mann-Whitney *U* test (mean rank) for comparing the density in saliva of bacteria from the orange and red complex among children and adolescents from the G-DS and G-ND in age groups 3–7 years and 8–12 years.

Bacteria		3–7 years			8–12 years		
		G-DS	G-ND	*p-value*	G-DS	G-ND	*p-value*
Orange complex	*C*. *rectus*	15.83	12.10	0.249^ns^	20.79	13.93	0.045*
	*F*. *nucleatum*	17.03	9.95	0.029*	19.42	14.75	0.173 ^ns^
	*P*. *intermedia*	18.44	7.40	0.001*	20.04	14.38	0.098 ^ns^
	*P*. *nigrescens*	17.69	8.75	0.006*	19.67	14.60	0.138 ^ns^
Red complex	*T*. *forsythia*	13.47	16.35	0.375^ns^	17.00	16.20	0.815 ^ns^
	*P*. *gingivalis*	16.39	11.10	0.103^ns^	19.71	14.58	0.133 ^ns^
	*T*. *denticola*	15.78	12.20	0.270^ns^	19.88	14.48	0.114 ^ns^

* Significant difference—Mann-Whitney *U* test.

ns—Not significant difference—Mann-Whitney *U* test.

## Discussion

This study was conducted to quantitatively and qualitatively evaluate the periodontopathic bacteria that are considered etiologic agents for the establishment of periodontal disease in saliva samples from children and adolescents with Down syndrome. The makeup of both groups was similar in terms of age and gender. The abilities of children and adolescents to understand and cooperate were among the inclusion criteria because clinical examination and saliva collection could be particularly difficult for individuals with Down syndrome who have severe intellectual disabilities.

The identification of salivary biomarkers for periodontal disease may help assess periodontal disease and the risk of developing periodontal disease. Previous studies have shown that bacterial testing in saliva is effective and may replace other well-established but more intricate methods of microbial analysis in periodontal disease [31]. Positive associations between the levels of periodontal pathogens in saliva and in subgingival plaque have been demonstrated, with strong correlations observed between *A*. *actinomycetemcomitans* (78.95%), *P*. *gingivalis* (86.84%), *T*. *denticola* (94.74%), *T*. *forsythia* (96.05%), *C*. *rectus* (85.53%), *P*. *intermedia* (85.53%), *F*. *nucleatum* (98.68%) and *P*. *nigrescens* (67.11%) in the salivary and subgingival plaque samples from patients with chronic periodontitis and aggressive periodontitis [32]. Salivary testing for periodontopathic bacteria is based on the idea that whole saliva and periodontal lesions tend to harbor similar relative levels of periodontal pathogens. High salivary levels of periodontal pathogens imply the presence of periodontitis or the risk of developing periodontitis; a decrease in the number of periodontal pathogens in the saliva can be used to assess the effectiveness of therapeutic intervention [33]. Because saliva collection is a simple, safe and non-invasive method, it should be a suitable technique for evaluating children and adolescents.

The periodontal status results showed that the DS group had a significantly higher percentage of participants with periodontal inflammation, as estimated by the gingival bleeding index; the data were consistent with previous studies that revealed a susceptibility to disease among children and adolescents with Down syndrome [2–6]. The high prevalence and severity of gingival bleeding can be attributed to factors such as immunodeficiency [3,4], elevated and early colonization by periodontopathic bacteria [2,5,6] and motor difficulty in performing oral hygiene, with a subsequent accumulation of plaque [3,7,13]. However, the mean plaque index in the present study was low and was similar in both groups, thus corroborating previously reported data [4]. This finding is probably because personal oral hygiene care was performed by the parents or guardians of DS children and adolescents.

Although differences in periodontopathic microbiota may explain the higher susceptibility to periodontal disease that is often observed in individuals with DS, few studies have described the bacterial species associated with this disease in young individuals. In the current study, microbiological evaluation showed a high prevalence (80–100%) of the eight species of periodontopathic bacteria in the total sample. In samples from participants in the G-DS, the prevalence ranged from 96.7% (*C*. *rectus*, *P*. *gingivalis*, *T*. *denticola*, *F*. *nucleatum* and *P*. *intermedia*) to 100% (*A*. *actinomycetemcomitans*, *P*. *nigrescens* and *T*. *forsythia*); these rates were higher than those reported in previous studies [2,5,6,12,13]. However, considering the number of bacterial cells of each species, children and adolescents with DS exhibited a higher percentage of microorganisms present in saliva compared to participants in the ND group. This result suggests that in addition to the presence of other bacteria, the number of cells of each type of bacteria is different in the ND group.

These differing results can be attributed to the methods used; to the best of our knowledge, this is the first study to use the FISH technique to detect, identify and quantify periodontal bacteria in the saliva of individuals with DS. Culture-based methods are time consuming and too selective; therefore, this approach does not reflect the exact composition of mixed bacterial communities or the microbial diversity in infections [34]. Molecular techniques such as PCR do not provide information about morphology, cell number, spatial distribution or the cellular environment of the organisms. Because of the paucity of morphological distinctions, microscopic analyses of bacteria do not provide reliable identification. This obstacle can be overcome by immunofluorescence using species-specific monoclonal antibodies. This technique is often hampered by unspecific binding, and it depends heavily on phenotypic antigen variation and expression. In addition, the target microorganism must be cultured first to raise a specific antibody [35]. Moreover, cross-reactivity was reported as a severe problem in the application of monoclonal antibodies to study complex microbiota. In contrast, FISH combines the precision of molecular genetics with visual information from microscopy to permit the visualization and identification of individual microbial cells within their natural microhabitat or diseased tissue [14].

This technique provides direct quantitative results without a prior culture; the visualization and quantification of individual microbial cell density can be performed quickly and objectively [14,15,27]. The use of the FISH technique permitted the observation of significantly higher densities of *C*. *rectus*, *P*. *gingivalis*, *T*. *denticola*, *F*. *nucleatum*, *P*. *intermedia* and *P*. *nigrescens* in the DS group. These results are similar to those of previous studies that used other methods to identify periodontal microorganisms in children and adolescents with DS compared to a control group. It has been suggested that early periodontopathic bacteria colonization in children with Down syndrome results in an alteration of the oral cavity microbiota and consequently leads to the development of periodontal disease at younger ages [5,6,12].

No significant difference between groups was found in *A*. *actinomycetemcomitans* densities, similar to previously published results [2]. However, other authors have shown a relationship between increased colonization by this species and a higher frequency of periodontal change in children and adolescents with Down syndrome [5,6,12,13]. The possibility of *A*. *actinomycetemcomitans* being detected in healthy periodontal sites has been previously suggested [2,8] and may explain the results obtained in the ND group, which showed a lower frequency of bleeding sites. These findings suggest that the role of *A*. *actinomycetemcomitans* in the early establishment of periodontal disease in individuals with DS is not fully understood.

A significant result in this study is the significantly higher density of bacteria from the orange complex in participants between the ages of 3 and 7 years in the DS group, (*F*. *nucleatum*, *P*. *intermedia* and *P*. *nigrescens*), which indicates the increased colonization of periodontal bacteria in younger children. Bacteria from the orange complex precede and prepare the environment for the colonization of microorganisms from the red complex, including those that are considered the main etiological agents of periodontal disease. These bacteria are associated with the presence of bleeding on probing [11], which is observed at a higher frequency in the DS group. Evaluation by age provides a theory of ecological succession and, consequently, the replacement of the species. Although no significant difference between the groups for bacteria from the red complex was observed according to age group, only *T*. *forsythia* did not differ significantly when an evaluation was performed without division according to age. Thus, the presence of bacteria from the orange complex may have favored the higher colonization of two of the three bacteria from the red complex (*P*. *gingivalis* and *T*. *denticola*).

The results of the current study indicate that children and adolescents with Down syndrome have a higher susceptibility to periodontal changes and a higher prevalence and density of some periodontal pathogens. These results are consistent with the idea that in these subjects, certain periodontopathic bacteria and specific associations among certain species may contribute to the increased prevalence and severity of periodontal disease [36]. These findings raise questions about the reasons for the elevated bacterial colonization in children and adolescents with DS. The data obtained in the current study add important information regarding the microbiota of periodontal disease in children and adolescents with Down syndrome. These data support regular periodontal examinations for the early diagnosis of periodontal disease establishment and the implementation of preventive measures tailored to the specific needs of these individuals.

## Supporting Information

S1 Table Chi-square test to verify the significance of the association between groups (G-DS and DN) and gingival bleeding index. (PDF)

S2 Table T-test to verify the significance of the association between groups (G-DS and DN) and Plaque index. (PDF)

S3 Table Descriptive measures (mean, median, minimum, maximum and standard deviation) for comparison of bacterial density (cells/mL X 10^8^) in the saliva of children and adolescents from G-DS and G-ND. (PDF)

S4 Table Results of Mann-Whitney *U* test (mean rank and sum of ranks) for comparison of bacterial density (cells/mL X 10^8^) in the saliva of children and adolescents from G-DS and G-ND. (PDF)
