# Supplementary material for: Salivary Periodontopathic Bacteria in Children and Adolescents with Down Syndrome
Source: PLoS One. 2016 Oct 11;11(10):e0162988. doi: 10.1371/journal.pone.0162988 (PMC5058504; doi:10.1371/journal.pone.0162988)
Supplement: S1 Table — (PDF) [file pone.0162988.s001.pdf]

SUPPORTING INFORMATION

SUPPLEMENTARY TABLES

Supplementary Table 1 - Table S1: Chi-square test to verify the significance of the association between groups (G-DS and DN) and gingival bleeding index

Chi-Square Test					
	Value	Df	Asymp. Sig (2-sided)	Exact Sig. (2-sided)	Exact Sig. (1-sided)
Pearson Chi-squar	4.356[b]	1	0.37		
Continuity correctione[a]	3.200	1	0.74		
Likeihood ration	4.490	1	0.34		
Fisher's exact test				0.72	0.36
linear-by-linear association	4.283	1	0.38		
N of valid cases	60	1			

[a] Computed only for a 2x2 table

[b] 0 cells (0%) have expected count less than 5. The minimun expected count is 7.5
