# Supplementary material for: Salivary Periodontopathic Bacteria in Children and Adolescents with Down Syndrome
Source: PLoS One. 2016 Oct 11;11(10):e0162988. doi: 10.1371/journal.pone.0162988 (PMC5058504; doi:10.1371/journal.pone.0162988)
Supplement: S2 Table — (PDF) [file pone.0162988.s002.pdf]

SUPPORTING INFORMATION

SUPPLEMENTARY TABLES

Supplementary Table 3 - Table S3: Descriptive measures (mean, median, minimum, maximum and standard deviation) for comparison of bacterial density (cells/mL X 10^8) in the saliva of children and adolescents from G-DS and G-ND

Group		CARE	B/TAFO	POGI	TREII	FUS	PINT	PNG	ACAC	DAPI	Som of the probes	% do total
SD	N	30	30	30	30	30	30	30	30	30	30	30
	Mean	17.707	17.830	13.140	12.7650	19.335	19.710	19.455	12.1350	1082.3333	132.08	0.121687
	Median	13.500	12.550	10.800	10.1250	11.700	12.150	9.900	8.7750	1052.5500	107.10	0.098641
	Minimum	5.0	0	0	0	0	0	1.4	1.35	492.75	31	0.0261
	Maximum	68.4	76.5	50.4	37.35	83.7	82.8	107.1	40.05	1489.05	357	0.2715
	Std. Deviation	14.3850	18.3105	9.4396	9.30328	22.4682	18.1477	22.9224	8.8375	267.5856	88.069	0.066028
NSD	N	30	30	30	30	30	30	30	30	30	30	30
	Mean	15.038	20.865	10.905	12.0233	23.760	12.660	18.240	16.3200	1215.0000	129.81	0.101393
	Median	6.750	14.400	5.175	3.6000	4.725	3.600	4.050	5.8500	1148.6250	70.88	0.052354
	Minimum	0	2.7	0	0	0	0	0	0	878.40	12	0.0108
	Maximum	76.1	57.6	77.0	85.50	245.7	90.9	98.1	85.05	2282.40	518	0.3988
	Std. Deviation	21.1195	17.8565	15.7386	19.73584	50.6749	21.5786	29.0168	24.24590	262.2316	139.392	0.099813
Total	N	60	60	60	60	60	60	60	60	60	60	60
	Mean	16.373	19.348	12.023	12.3942	21.548	16.185	18.848	14.2275	1148,6667	130.94	0.111540
	Median	11.600	12.800	9.450	8.5500	7.875	9.900	8.775	7.2000	1127.2500	88.60	0.087650
	Minimum	0	0	0	0	0	0	0	0	492.75	12	0.0108
	Maximum	76.1	76.5	77.0	85.50	245.7	90.9	107.1	85.05	2282,40	518	0.3988
	Std. Deviation	17.965	17.996	12.915	15.3013	38.927	20.084	25.932	18.215	271.051	115.603	0.084525
