# Supplementary material for: Salivary Periodontopathic Bacteria in Children and Adolescents with Down Syndrome
Source: PLoS One. 2016 Oct 11;11(10):e0162988. doi: 10.1371/journal.pone.0162988 (PMC5058504; doi:10.1371/journal.pone.0162988)
Supplement: S3 Table — (PDF) [file pone.0162988.s003.pdf]

SUPPORTING INFORMATION

SUPPLEMENTARY TABLES

Supplementary Table 4 - Table S4: Results of Mann-Whitney *U* test (mean rank and sum of ranks) for comparison of bacterial density (cells/mL X 10^8) in the saliva of children and adolescents from G-DS and G-ND

	CARE	B/TAFO	POGI	TREII	FUS	PINT	PNG	ACAC	DAPI	Sum of probes	% total
Mann-Whitney U	281.500	413.000	298.500	299.500	281.500	228.500	270.000	321.500	336.000	335.000	309.000
Wilcoxon W	746.500	878.000	763.500	764.500	746.500	693.500	735.000	786.500	801.000	800.000	774.000
Z	-2.493	-0.547	-2.241	-2.228	-2.493	-3.277	-2.664	-1.901	-1.685	-1.700	-2.085
Asymp. Sig. (2-tailed)	0.013	0.584	0.025	0.026	0.013	0.001	0.008	0.057	0.092	0.089	0.037

a Grouping Variable: group
