# Supplementary material for: Salivary Periodontopathic Bacteria in Children and Adolescents with Down Syndrome
Source: PLoS One. 2016 Oct 11;11(10):e0162988. doi: 10.1371/journal.pone.0162988 (PMC5058504; doi:10.1371/journal.pone.0162988)
Supplement: S4 Table — (PDF) [file pone.0162988.s004.pdf]

SUPPORTING INFORMATION

SUPPLEMENTARY TABLES

Supplementary Table 2 - Table S2: T-test to verify the significance of the association between groups (G-DS and DN) and Plaque index

Independent Samples Test									
	Levene's Test for Equality of Variances		T-test for Equality of Means						
	F	Sig.	t	Df	Sig. (2-tailed)	Mean difference	Std. Error difference	95% confidence interval of the difference	
IP								Lower	Upper
Equal variances assumed	0.182	0.671	0.642	58	0.523	0.7333	0.1142	-0.155	0.302
Equal variances not assumed			0.642	58	0.523	0.7333	0.1142	-0.155	0.302
